# Late complication of a renal calculus: fistulisation to the psoas muscle, skin and bronchi

**DOI:** 10.1590/S1677-5538.IBJU.2014.0541

**Published:** 2015

**Authors:** Ziga Snoj, Nenad Savic, Jaka Regvat

**Affiliations:** 1 Radiology Institute, University Medical Centre, Zaloška 2, Ljubljana, Slovenia

## Abstract

Kidney disease presenting with cutaneous fistula is a rare condition. We present a case of a 90-year-old woman with dementia who had no prior urological problems and had a cutaneous fistula in the left lumbar region. A fistulogram and computer tomography examination revealed a large staghorn calculus with signs of xanthogranulomatous pyelonephritis in the left kidney and renal fistulisation to the psoas muscle, skin and bronchi. To our knowledge this is the first report in the literature of coexisting renal fistulisation to the psoas major muscle, skin and bronchi. This report illustrates how computed tomography in combination with fistulography can resolve the diagnostic dilemma that pertains to the complex spread of the disease in cases involving nephrocutaneous fistula. Furthermore, the report shows how a renal calculus, even asymptomatic, can cause a serious medical condition, and highlights the importance of early medical intervention.

## INTRODUCTION

Retroperitoneal and psoas muscle abscesses are common complications in renal disease. Fistula formation between the kidney and its adjacent organs has been reported in the literature due to a number of causes including surgical complications, infection, trauma and stone disease (1). However, nephrocutaneous fistula (NCF) and nephrobronchial fistula (NBF) are rare manifestations in renal disease. To our knowledge this is the first report in the literature of coexisting renal fistulisation to the psoas muscle, skin and bronchi.

## CASE REPORT

A 90-year old woman with dementia was admitted to our hospital from a nursing home with a cutaneous fistula in her left lumbar region with purulent discharge over the past month. We found out from the patient's daughter that the patient had had the fistula for at least one year but due to the patient's good general condition no diagnostic procedures were conducted. In the last few days prior to admission the daughter noticed that the patient was getting weaker, had no appetite, had a dry cough and was unable to walk due to severe left-sided back pain and coxalgia. The patient had no history of urological problems. On admission the patient was subfebrile (37.7°C) with no evident respiratory distress. Laboratory examination showed anaemia (haemoglobin, 8.7g/L), leukocytosis (17.1×109/L) and a raised CRP value (220mg/L). The results of the kidney function tests were normal.

**Figure 1:**
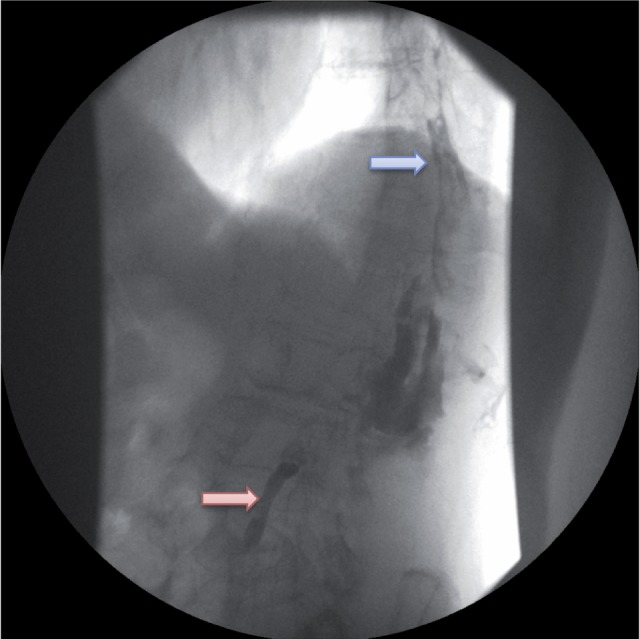
Fistulogram showing nephrocutaneous fistula with contrast leakage cranially (blue arrow) and caudally (red arrow) from the kidney.

The fistulogram revealed nephrocutaneous fistula with contrast leakage cranially and caudally from the kidney (Figure-1). For the purposes of carrying out a detailed examination we decided to conduct a computer tomographic (CT) scan immediately after the fistulogram.

**Figure 2:**
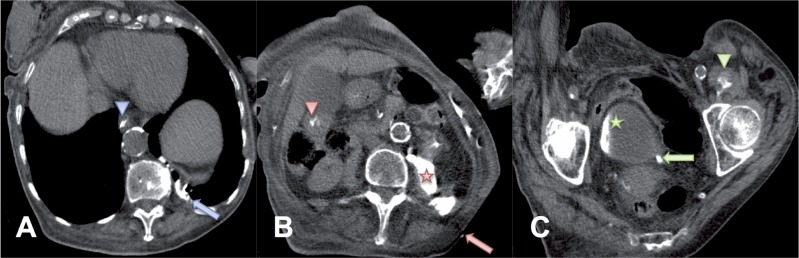
The computer tomographic scan performed immediately after the fistulogram; the patient was positioned on her right flank. A) contrast in the oesophagus (blue arrowhead) and communication with the left lower lobe of the lung (blue arrow). B) cutaneous fistula (red arrow), contrast retroperitoneally (red star) and in the duodenum (red arrowhead). C) contrast in bladder (green star), ureter (green arrow) and in the psoas muscle (green arrowhead) just proximally to lesser trochanter.

The patient was positioned on her right flank during the CT examination because of severe left sided coxalgia. NCF and NBF were observed on the CT (Figures 2 A and B). NCF was communicating with the renal pelvis and contrast was excreted into the bladder (Figure-2 C). NBF was communicating with the bronchi of the posterobasal segment of the left lower lobe through the lumbocostal trigone of the diaphragm (Figure-2 A). Contrast was observed in the left main bronchus and trachea. Furthermore, contrast was also observed in the oesophagus, stomach and duodenum after expectoration from the bronchial tree (Figures 2 A, B and C). Caudal contrast leakage was shown retroperitoneally along the whole length of the psoas and the iliacus muscle, all the way to the lesser trochanter (Figure-2 C). A morphologically deformed kidney was observed.

**Figure 3:**
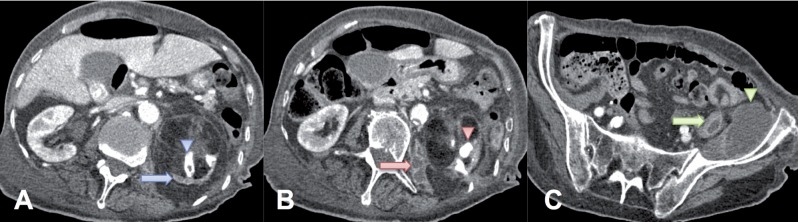
The computer tomographic scan with intravenous contrast. A) staghorn calculus (blue arrowhead) and extremely atrophic parenchyma of the left kidney (blue arrow). B) retroperitoneal abscess (red arrow) and staghorn calculus (red arrowhead). C) fistulisation along the psoas muscle (green arrow) and large pelvic abscess (green arrowhead).

Detailed examination of the deformed kidney and the caudal contrast leakage was performed with a follow-up CT scan five days later with intravenous contrast application. A large staghorn calculus was observed within the collecting system of the enlarged left kidney (Figures 3 A and B). Non-enhancing areas of the dilated collecting system, surrounded by enhancing and extremely atrophic parenchyma, were observed in the left kidney–a finding known as the “bear paw” sign (Figure-3 A). These CT findings of the deformed left kidney are typical for xanthogranulomatous pyelonephritis. The caudal contrast leakage was found to be a retroperitoneal abscess fistulising along the psoas muscle and the iliacus muscle, forming a large pelvic abscess (Figure-3 C). The right kidney was normal.

E. coli grew from fistular smear and urine cultures. The QuantiFERON test was negative. The inflammatory markers were found to be depleting after the initial empirical therapy with fucloxacillin and ciprofloxacin that were changed to specific antibiotic coverage with piperacillin and tazobactam. Considering the general condition and extent of the disease, the patient was not a suitable candidate for surgical treatment and was managed conservatively.

## DISCUSSION

We report on a unique case of triple renal fistulisation. To our knowledge this is the first report in the literature with fistulisation to the psoas muscle, skin and bronchi as a late complication of a renal calculus.

A psoas muscle abscess is a relatively rare condition with renal disease being the cause of abscess formation in 17.5% of cases (2,3). Spontaneous fistulisation to the adjacent organs due to infectious or inflammatory disease is not uncommon. However, fistulisation to the skin and bronchi without previous renal surgery is very rare. A Medline search revealed 16 cases of spontaneous NCF since 1983 and 24 cases of NBF since 1956. Furthermore, we found only two reported cases of coexistent NCF and NBF (4, 5) and none for coexisting fistulisation to the psoas muscle, skin and bronchi.

In the past, urinary tract tuberculosis was considered to be the primary cause of renal fistulisation. Nowadays spontaneous fistulas usually occur because of a chronic infection, inflammation, and typically in association with xanthogranulomatous pyelonephritis (XGP). These kidney diseases are generally associated with calculus formation (1, 6). Various microorganisms have been cultured from the sputum, urine and kidney specimens in patients with NCF and NBF (7). However, E. coli and Proteus species account for approximately one third of cases (8).

A final diagnosis of renal disease was not made since the patient was not a suitable candidate for surgery. A definite diagnosis of XGP is histological (9); however the CT scan of our patient was highly demonstrative of diffuse XGP showing a severely enlarged kidney and staghorn calculus. These two signs, along with the “bear paw” sign and inflammatory changes in the perinephric fat, although not specific, are strongly suggestive of diffuse XGP (9).

The kidney is a retroperitoneal organ and directly related to the muscles posteriorly. The only intervening layers are perinephric fat and Gerota's fascia. Once these are transgressed any progressive inflammatory or degenerative process involving the kidney can spread along the transversalis fascia to the posterior retroperitoneal space (10, 11). Fusion lines of fascial planes tend to direct the exudates within the retroperitoneal compartment and the inflammatory spread is usually caudal (12). However, in rare cases the spread can be cranial with the potential for forming NBF. Furthermore, the lumbocostal trigone is described as a relatively weak area of the diaphragm with the possibility of the transmission of infection to the thoracic cavity (13).

In this case the renal inflammatory process spread laterally, cranially and caudally from the posterior retroperitoneal space. Laterally it transgressed the transversalis fascia and formed NCF just laterally of the erector spinae muscle group (Figure-2 B). Cranially, the inflammatory process transgressed the transversalis fascia and formed NBF through the lumbocostal trigone (Figure-2 A). Caudally, the inflammatory process transgressed the transversalis and psoas fascia and spread along the psoas muscle, forming a large pelvic abscess (Figure-3 C).

There are several radiologic methods available for revealing the anatomical course of fistulae. In cases of renal fistulisation, a fistulogram and retrograde pyelography are usually the first step in the diagnostic procedure and are a good choice in cases where the spread of the disease is non-complicated (4, 5, 11, 14). However, in cases where the spread is complex, as observed in this case, a CT in conjunction with fistulography is the best method to establish a diagnosis.

## CONCLUSIONS

Our case report demonstrates the difficulties encountered in diagnosing this rare condition. Furthermore, it demonstrates how a neglected calculus, though asymptomatic, can cause a serious medical condition and shows the importance of early medical intervention.
